# Early Gut Microbiota Intervention Suppresses DSS-Induced Inflammatory Responses by Deactivating TLR/NLR Signalling in Pigs

**DOI:** 10.1038/s41598-017-03161-6

**Published:** 2017-06-12

**Authors:** Yi Xiao, Honglin Yan, Hui Diao, Bing Yu, Jun He, Jie Yu, Ping Zheng, Xiangbing Mao, Yuheng Luo, Daiwen Chen

**Affiliations:** Key Laboratory of Animal Disease-Resistance Nutrition, Animal Nutrition Institute, Sichuan Agricultural University, Yaan, Sichuan China 625014

## Abstract

Recent metagenomic studies suggest that innate and adaptive immune phenotypes can be programmed via gut microbiota-host interactions mediated via activation of pattern recognition receptors (PRRs) on host cells. In this study, we used two extremely different pig lines (the Yorkshire and the Tibetan) to test the hypothesis that the transplantation of gut microbiota could transfer certain immunologic characteristics from donor to recipient. The faecal microbiota of these two pig lines was transplanted in healthy commercial hybrid newborn piglets to establish the “Tibetan-intervened” and “Yorkshire-intervened” porcine models. Then, acute colitis was induced using dextran sulphate sodium (DSS), which activated Toll-/NOD-like receptor (TLR/NLR) signalling in the colonic tissues of the “Yorkshire-intervened” piglets, leading to increases in pro-inflammatory cytokines and immune cells and causing intestinal injuries. Conversely, DSS administration had little influence on the “Tibetan-intervened” piglets, which showed no significant inflammation and no changes in cytokines, immune cells, or signalling molecules, including TLRs, NLRs, MYD88 and NF-κB, after DSS treatment. These results indicate that pigs inoculated with the Tibetan microbiota acquired relatively strong resistance to experimental colitis, suggesting that the genotype of the host contributes to the uniqueness of its intestinal microbial community, whereas the microbiota plays a vital role in programming the immune phenotypes of the host.

## Introduction

Shaped by evolution over millions of years, some symbiotic bacteria have developed beneficial relationships with their hosts by creating a mutualistic environment in which the microbiota contributes to many host physiological processes and in turn is provided with essential conditions for survival by the host^1, 2^. Pattern recognition receptors (PRRs), including the Toll-like receptors (TLRs) and NOD-like receptors (NLRs), are receptor molecules that are expressed in immune cells. In innate immunity, PRRs play an important protective role against pathogens by recognizing pathogen-associated molecular patterns (PAMPs), such as flagellin and bacterial nucleic acid^3^. Moreover, the gut microbiota can modulate the expression of genes involved in inflammatory responses through PRRs^4^. TLRs recruit the signalling adaptors MYD88 and TIR-domain-containing adaptor-protein-inducing interferon-β (TRIF) for ligation to signalling molecules via nuclear factor-κB (NF-κB). This process enhances the production of various pro-inflammatory cytokines (i.e., IL-1β, IL-6 and TNF-α)^5^, which can potentially lead to the activation of classic pro-inflammatory signalling and contribute to host defence against pathogens. Greater inflammation can, however, result if the interactions between the PRRs and microbial products are blocked^6, 7^, thus underlining an intrinsic interplay between the microbiota and the host that may sustain tissue integrity during optimal conditions. Under normal conditions, TLR signalling activates negative regulators of both TLR and NLR signalling; thus, despite the enormous number of PRR ligands in the gut, the PRRs can be utilized to protect the gut without activating dangerous levels of inflammation^8, 9^. However, a delicate balance exists between the host immune system and the bacterial populations; and disruption of this balance may cause dysbiosis and even an inflammatory response in the host^3^. For example, inflammatory bowel disease (IBD) is closely associated with the immune response caused by the gut microbiota through PRR signalling. The primary evidence that IBD can result from an alteration in PRR-microbiota interactions is that disease in colitis models driven by adaptive immunity is ameliorated by the ablation of PRR signalling. For example, the loss of TLR signalling alleviates chronic T cell-mediated colitis related to microbial dynamics in multiple models^10, 11^, and MYD88-deficient mice are protected from spontaneous or induced colitis^12, 13^. Thus, PRR signals can not only sense pathogens and promote the induction of innate effectors but may also promote autoimmune diseases under inflammatory conditions. In both cases, the gut microbiota plays a key role in modulating the adaptive immune response.

Therefore, interesting questions have arisen regarding whether the gut microbiota is a regulator of health and disease and what constitutes a healthy microbiome. Recently, a growing understanding of the complexity of the gut microbiome has been achieved with high-throughput techniques, such as metagenomics and 16S rRNA-based approaches. Although this question still remains largely unanswered due to the uniqueness of the microbiota within each individual, especially at the species and strain levels^14^, studies have shown that host genetic factors clearly contribute to the individuality of the gut microbiota composition, which changes with genetic variations of the host^15, 16^. Nevertheless, recent metagenomic studies suggest that some bacterial genes may in practice be ‘phenocopies’ that are potentially functional and able to perform certain functions for the host^17^. This possibility raises the hypothesis that the gut microbiota may in turn contribute to individual phenotypes. One possible method to test this hypothesis is by faecal microbiota transplantation (FMT). The use of FMT has a very high success rate in curing colitis caused by *Clostridium difficile* ^18^. Another study colonized germ-free (GF) mice with a mouse microbiota or human microbiota and showed that the nature of the colonizing microbiota affected the host’s initial T cell populations^19^, providing further evidence for host-linked co-evolution of the microbiota and immune responses. These findings are paving the way for the use of the microbiome to improve human health in the near future; however, our understanding of microbial communities and their interactions with the host is still very limited. Understanding how microbes behave during FMT could be helpful for the selection of optimal microbial features of a transplanted microbiota to ensure a successful outcome. Therefore, the aim of the present study was to characterize the role of the microbiota in directing host immune responses using a distinctive porcine model based on two extreme pig strains (the Yorkshire and the Tibetan). The Yorkshire is a typical commercial pig breed that has been highly selectively bred to obtain a better growth rate and meat quality at the expense of reducing the pig’s disease resistance capacity. By contrast, the Tibetan strain is a domestic pig that is mainly distributed in the Tibetan highlands and has striking phenotypic and physiological differences from lowland pigs that have allowed them to adapt to the extreme conditions^20^. In this study, we compared (Part I) the active molecules related to TLR and NLR signalling and the faecal bacterial communities of these two pig strains using 16S rRNA sequence-based microbiome methods. Then, we colonized recipient animals (Yorkshire piglets) with the faecal microbiota from the Tibetan pigs. By tracking (Part II) the phenotypic expression, the PRR signalling pathways and the immune cells in the normal condition and acute colitis models (induced by dextran sulphate sodium), we show how immune traits can be regulated by altering the gut microbiota through FMT and how these donor and recipient animals can be used to conduct controlled proof-of-principle “clinical” metagenomic studies of host-microbiome interrelations.

## Results

### Part I: A comparative study of the faecal bacterial communities and active molecules related to TLR and NLR signalling in two pig strains

#### The composition of the faecal bacterial communities

**Figure 1:**
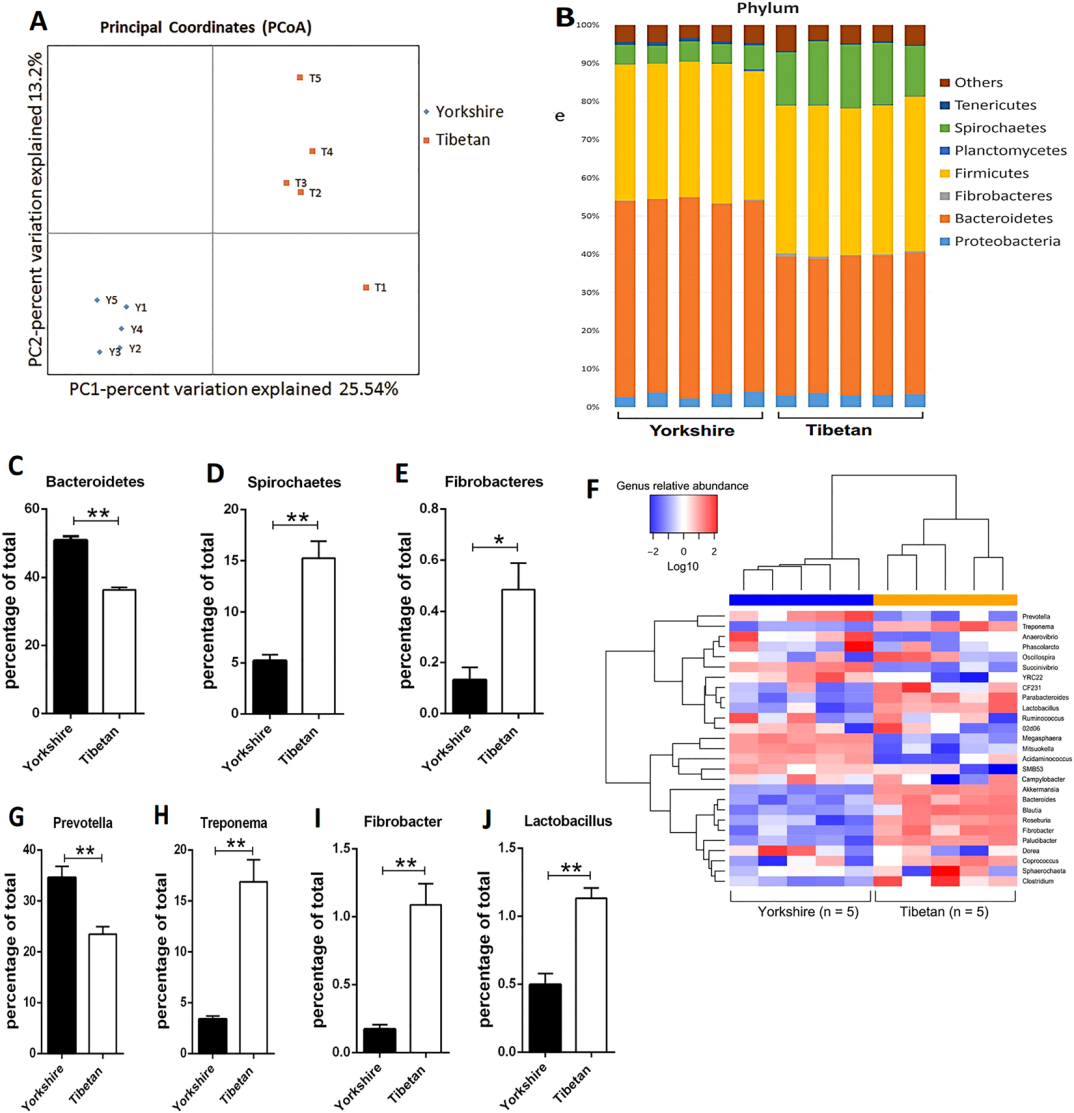
Analysis of the faecal microbiota of the Yorkshire and Tibetan pigs. (**A**) 16S rRNA gene surveys (analysed by unweighted UniFrac PCoA) of the faecal microbiota of the Yorkshire or Tibetan pigs (n = 10). PC1 and PC2 are shown on the x-axis and y-axis, respectively. The percentage of variance explained by each coordinate is shown in parentheses. (**B**) Relative abundance of the phyla in two pig strains (Yorkshire and Tibetan pigs). Groups within the same bacterial phylum are indicated by different shades of the same colour. Taxa with a mean relative abundance of >1% are shown (n = 5 for each strain). (**C**–**E**) Percentage of Bacteroidetes, Spirochaetes and Fibrobacteres in the faeces of Yorkshire and Tibetan pigs (each n = 5) in the classification of phylum. (**F**) Heatmap of log10-transformed abundance levels of the selected genera (>0.1% in at least one sample) for the individual Yorkshire and Tibetan faecal samples (each n = 5). The pigs with the highest and lowest bacterial levels are in green and red, respectively. (**G**–**J**) Percentage of *Prevotella*, *Treponema*, *Fibrobacter* and *Lactobacillus* in the faeces of Yorkshire and Tibetan pigs (each n = 5) in the classification of genus.

We performed a 16S rRNA gene-based survey of faecal specimens obtained from Yorkshire and Tibetan pigs, which consumed the same diet at all times. A total of 547,276 high quality sequences were obtained from 10 feacal samples (Table S1), with an average of 56,925 sequences per sample and a range of 40,134 to 60,492. The first two principal coordinates (representing 38.7% of the total variance) based on an unweighted UniFrac principal coordinate analysis (PCoA) (Fig. 1A) clearly separated the 10 faecal samples by genotype. Then, we examined the composition of the faecal microbiota of these two pig strains and found differences between them at each taxonomic level. At the phylum level (Fig. 1B), the most notable difference was that the relative abundance of the Bacteroidetes (Fig. 1C) was higher in the faeces of the Yorkshire pigs (FY) (50.8%) than in the faeces of the Tibetans (FT) (36.3%). The abundances of Spirochaetes (15.3% versus 5.2%, Fig. 1D) and Fibrobacteres (0.13% versus 0.49%, Fig. 1E) in FT were higher than the abundances in FY. Similarly, the microbial distributions at the genus level (Fig. 1F) showed more *prevotella* (Bacteroidetes phylum) in FY (34.6%) than in FT (23.5%) (Fig. 1G). The abundance of *Treponema* (Spirochaetes phylum, 16.9% versus 3.4%), *Fibrobacter* (Fibrobacteres phylum, 1.09% versus 0.17%) and *Lactobacillus* (1.13% versus 0.49%) was much higher in FT than in FY (Fig. 1H–J). Moreover, differences in the microbial distributions were found at other classifications from class to genus are shown as bar charts in Fig. S1.

#### Cytokine levels

**Figure 2:**
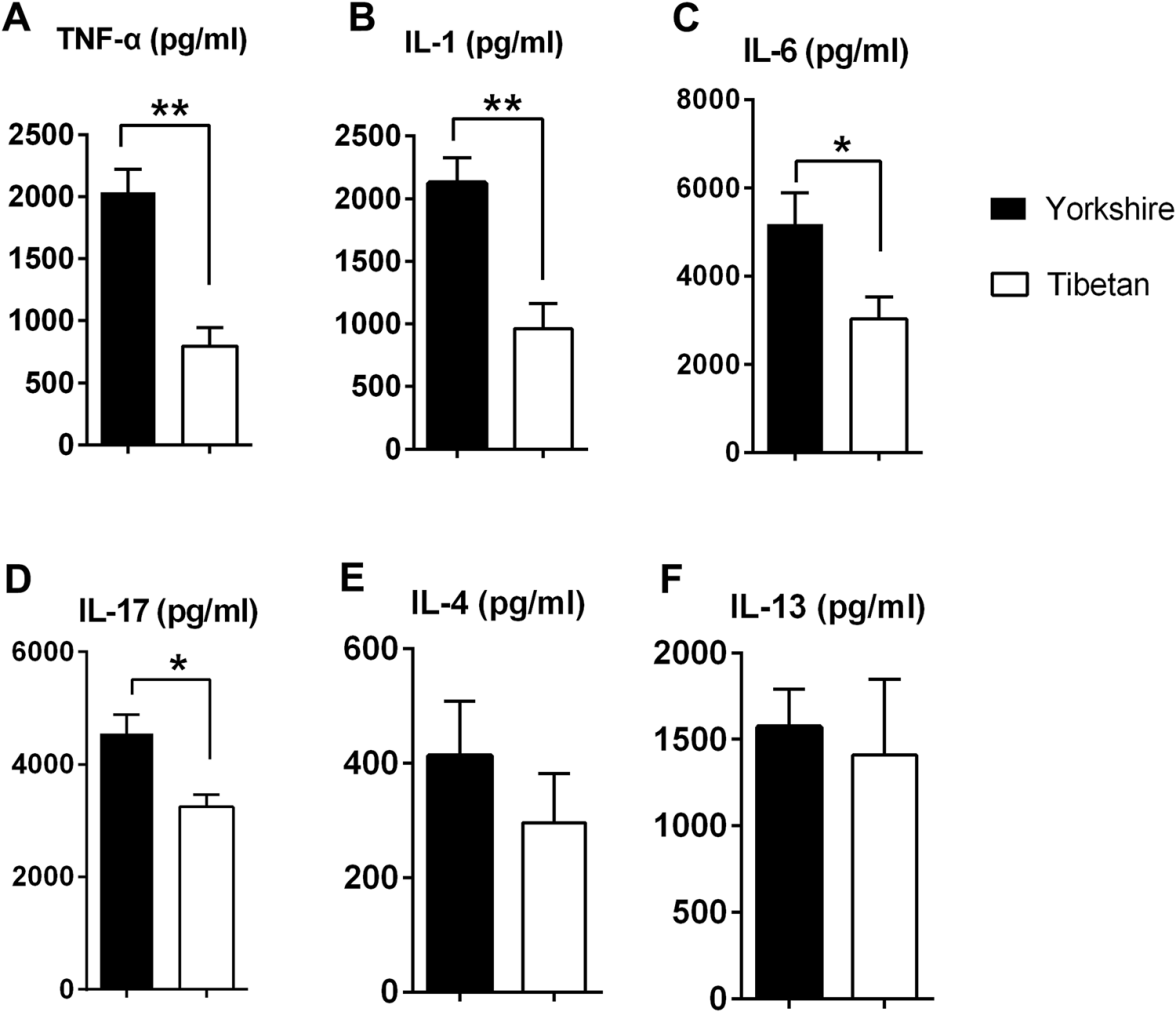
Quantitation of cytokines in the colon tissues by ELISA. The results are presented as the mean ± the SEM. Statistical significance was assessed by performing a Student’s t-test. *P < 0.05; **P < 0.01. Each n = 5.

The expression levels of several pro-inflammatory cytokines (IL-1β, IL-4, IL-6, IL-13, IL-17 and TNF-α) associated with TLR/NLR signalling were evaluated in colon samples from Yorkshire and Tibetan pigs using commercially available ELISA kits. The Yorkshire pigs showed higher intestinal levels of the measured pro-inflammatory cytokines TNF-a, IL-1β, IL-6 and IL-17 than the Tibetan pigs (Fig. 2). However, no differences in the levels of the gut homeostatic Type 2 related cytokines IL-4 and IL-13 were observed in the intestinal samples.

#### mRNA expression levels of TLRs, NLRs and associated molecules in intestinal tissues

**Figure 3:**
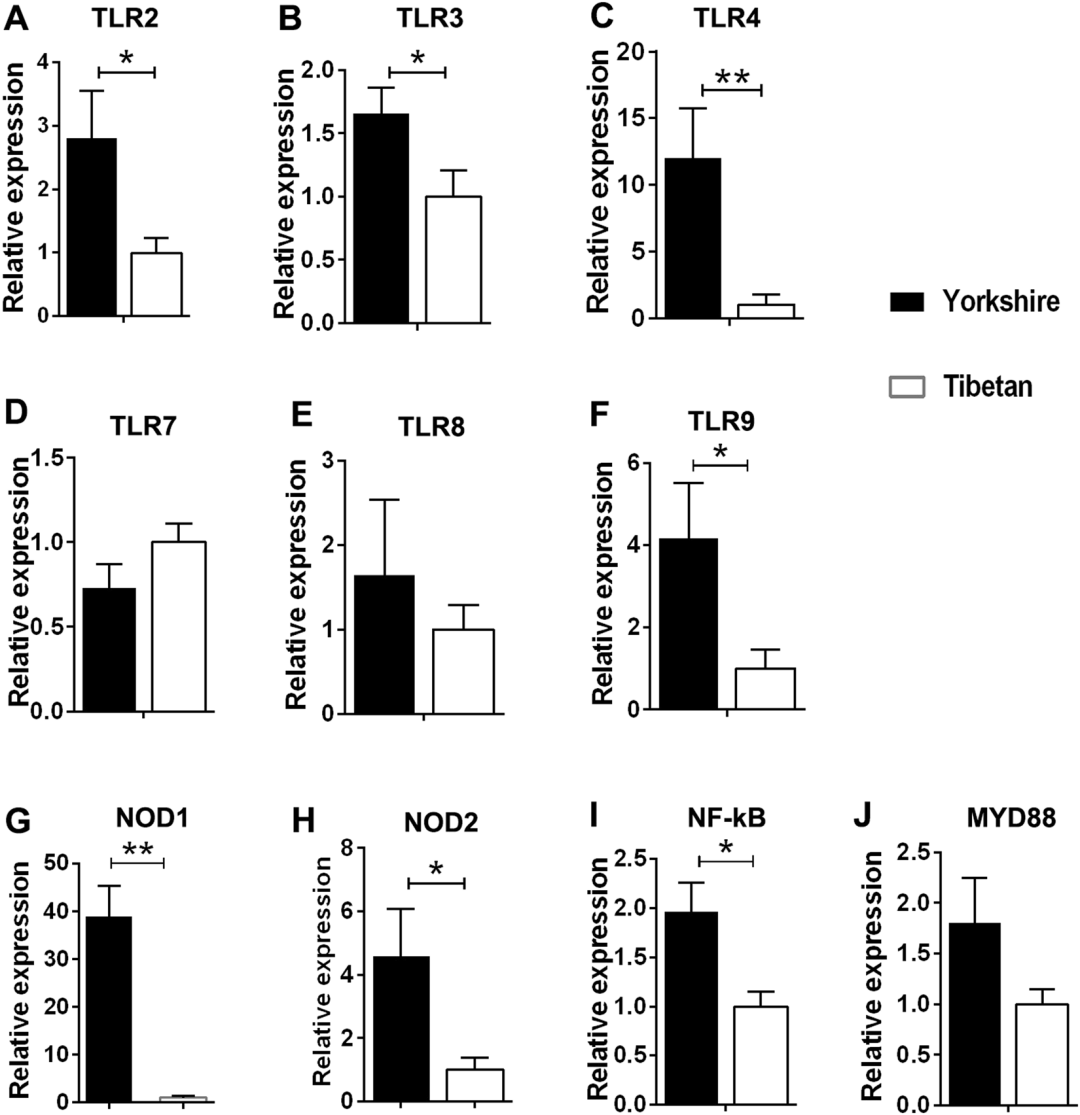
Relative quantities of mRNAs encoding TLRs, NLRs and associated molecules in the intestinal tissues of the Yorkshire and Tibetan pigs. Total RNA was prepared from the colon tissues of these two pig strains as described in the “Materials and Methods”. Relative mRNA expression was quantified by qRT-PCR. The results are presented as the mean ± the SEM. Statistical significance was assessed by performing a Student’s t-test. *P < 0.05; **P < 0.01. Each n = 5.

The mRNA expression levels of various PRRs and associated molecules were evaluated in colonic tissues from the two pig lines by qRT-PCR. The relative amounts of the mRNAs in each tissue are shown in Fig. 3. The abundances of the NLR (NOD1 and 2) gene transcripts were considerably higher in the Yorkshire pigs than in the Tibetan pigs. In particular, the number of NOD1 transcripts was more than 30-fold higher in the Yorkshire pigs. Similarly, most of the analysed TLR mRNAs were expressed at higher levels in the guts of the Yorkshire pigs (Fig. 3) with the exceptions of TLR7 and TLR8. We tracked the downstream regulators of TLR/NLR signalling; the results showed that the NF-κB mRNA level detected in the colons of the Tibetan pigs was lower than that detected in the Yorkshire pigs, but we only observed a trend for higher MYD88 expression in the Yorkshire pigs (*P* = 0.09).

### Part II: Effect of the symbiotic bacteria intervention on immune traits and resistance to colitis induction

In this part, the faecal microbiota from donor Yorkshire and Tibetan pigs was transplanted in healthy newborn piglets to establish the “Tibetan-intervened” and “Yorkshire-intervened” porcine models (see Materials and Methods for details).

#### Clinical activity and histology after DSS administration

**Figure 4:**
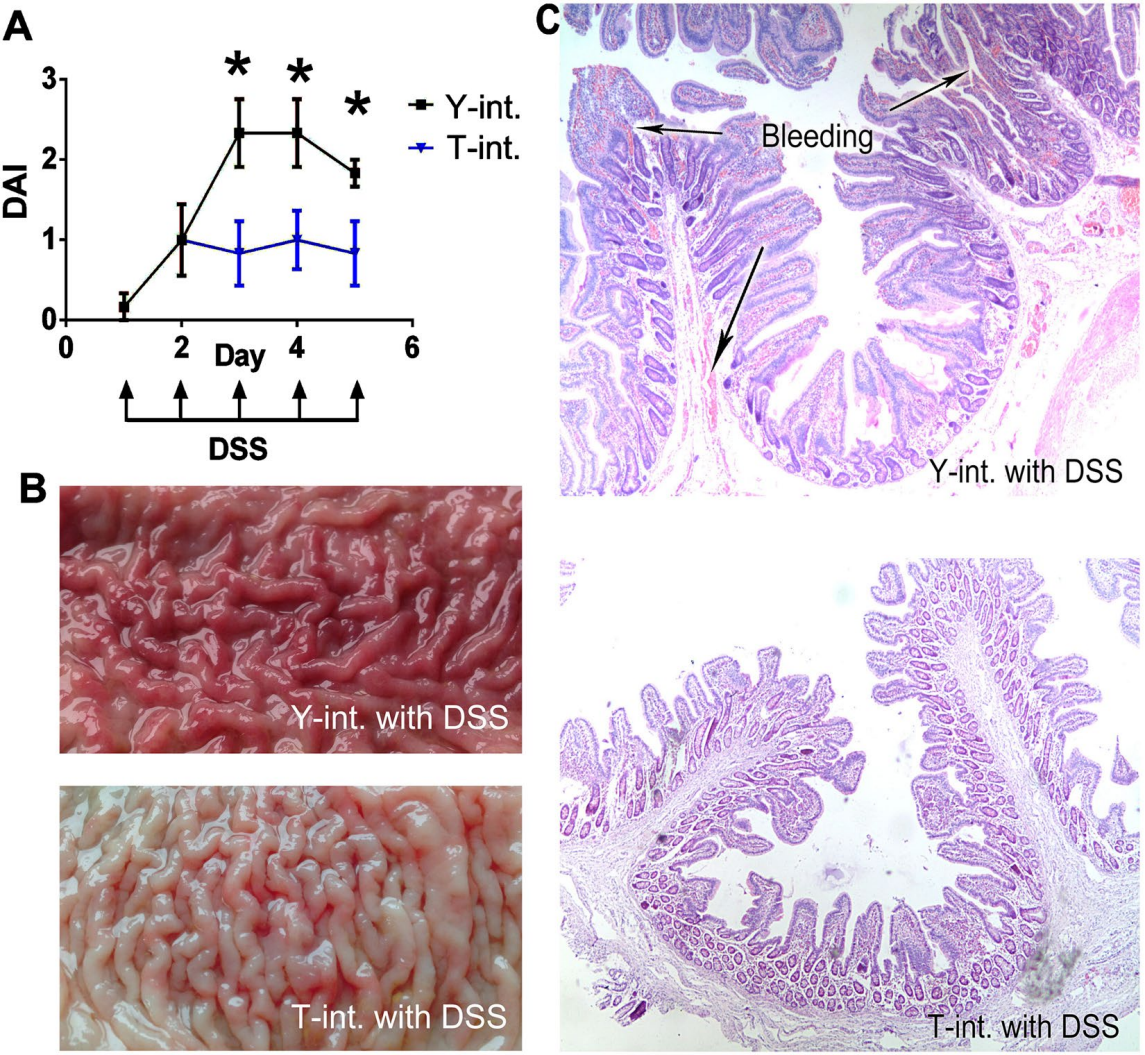
Treatment with the faecal microbiota from Tibetan pigs suppresses experimental colitis in the porcine models. The faecal microbiota of the Yorkshire and Tibetan pigs was transplanted in healthy commercial hybrid newborn piglets to establish the “Yorkshire-intervened” (Y-int.) and “Tibetan-intervened” (T-int.) porcine models. (**A**) Effect of the gut microbiota intervention on the disease activity index (DAI) in Y-int. and T-int. pigs treated with DSS (each n = 6). (**B**) Gross morphology of the colonic mucosal surface after 5 days of DSS administration. (**C**) Haematoxylin and eosin staining of the colon after 5 days of DSS administration (original magnification, x40). *P < 0.05.

Oral DSS administration induced observable acute colitis only in the pigs colonized with the “Yorkshire microbiota”. On days 3, 4 and 5 post-DSS induction, this group showed higher clinical activity, characterized by the disease activity index (DAI), than the pigs colonized with the “Tibetan microbiota” (Fig. 4A). Both the representative colonic macroscopic morphology (Fig. 4B) and histopathology (Fig. 4C) of the Yorkshire group showed a more severe degree of bleeding than the Tibetan group. The pigs in the control group showed no body weight loss and no blood in the stools during the entire induction.

#### Identification of related immune cells in colonic tissues

**Figure 5:**
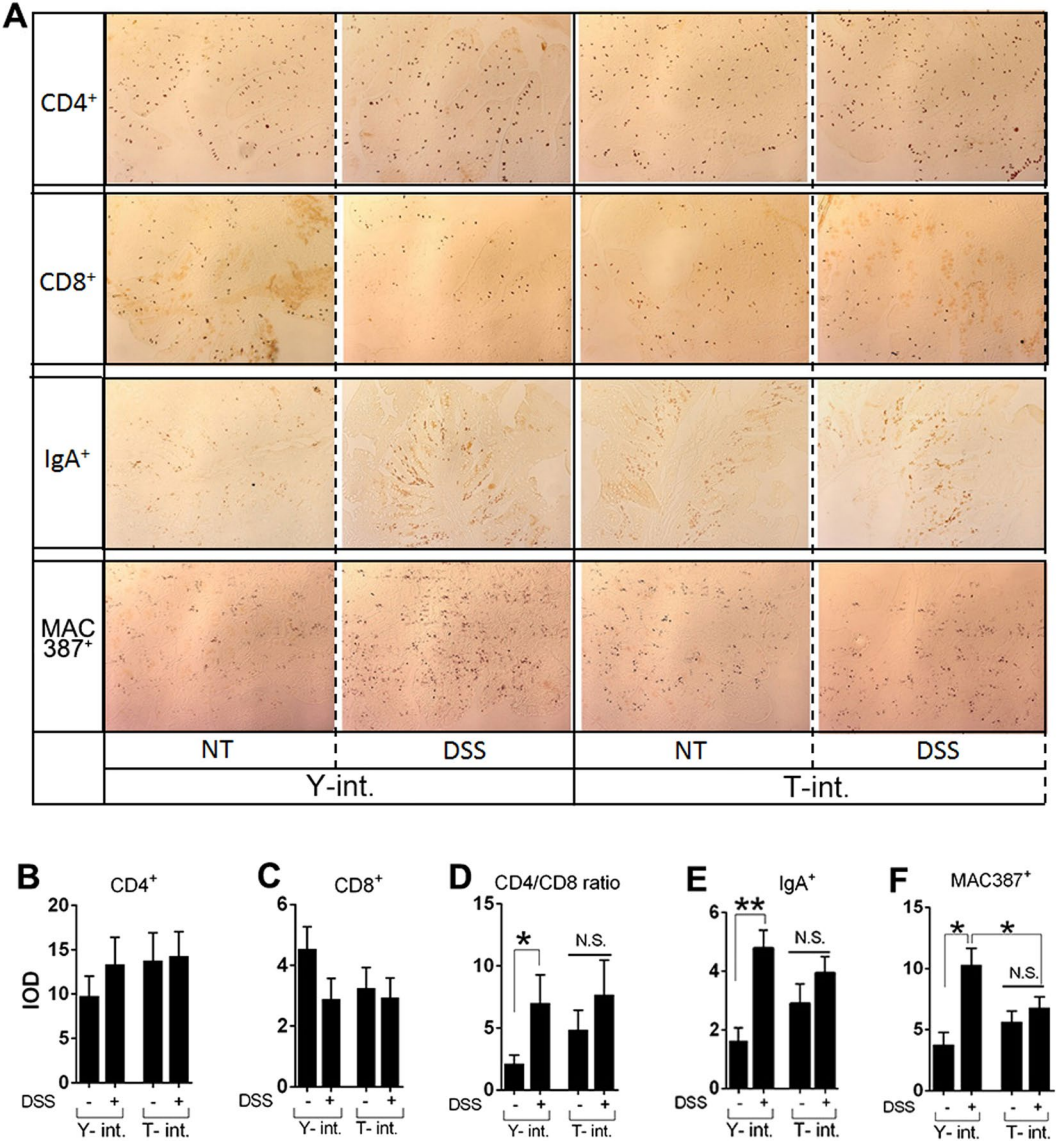
Immune cells in transplanted pigs was evaluated by Immunohistochemistry. (**A**) Immunohistochemistry to evaluate CD4^+^ T cells, CD8^+^ T cells, IgA^+^ plasma cells and MAC387^+^ macrophages. (**B**–**F**) Quantitative analyses of these positive reactions are presented in the bar graphs (n = 6 for each group). The intensity of staining was quantified using the Image-Pro Plus software. Representative graphs with integrated optical density (IOD) readings are expressed as arbitrary units (AU) ± the SEM. *P < 0.05; **P < 0.01. N.S., no significant difference.

An immunohistochemical analysis was performed on dewaxed colonic sections from 6 piglets of each FMT group. CD4^+^ T cells, CD8^+^ T cells, IgA^+^ plasma cells and MAC387^+^ macrophages were immunolabeled and formed granular brown reaction products in the colon tissues from the piglets (Fig. 5A). Control tissues (not shown) did not exhibit immunostaining with these antibodies. The semi-quantitative evaluation results are shown in Fig. 5B. In the Yorkshire group but not the Tibetan group, DSS infusion significantly increased the CD4^+^/CD8^+^ ratio, the number of IgA^+^ plasma cells and the number of MAC387^+^ macrophages in the colonic tissues. Compared with colonization with the “Yorkshire microbiota”, colonization with the “Tibetan microbiota” even decreased the number of macrophages induced by DSS in the colon. In the two groups without DSS treatment, the faecal transplant itself had no effect on these immune responses. These results indicated that the “Tibetan microbiota” was better equipped to reduce the sensitivity to DSS in the pig intestine than the “Yorkshire microbiota” and that it was better able to suppress immune responses, such as macrophage recruitment. We also stained tissues from the control group (without DSS and FMT); the levels of the above immune cells in the control group were approximately the same as the levels in the Yorkshire group without DSS administration (not shown). Moreover, the two-way ANOVA results showed a significant interaction between DSS treatment and FMT only for the CD4^+^/CD8^+^ ratio (*P* = 0.04, not shown in the graphs).

#### Cytokines and chemokines related to TLR and NLR signalling in colonic tissues

**Figure 6:**
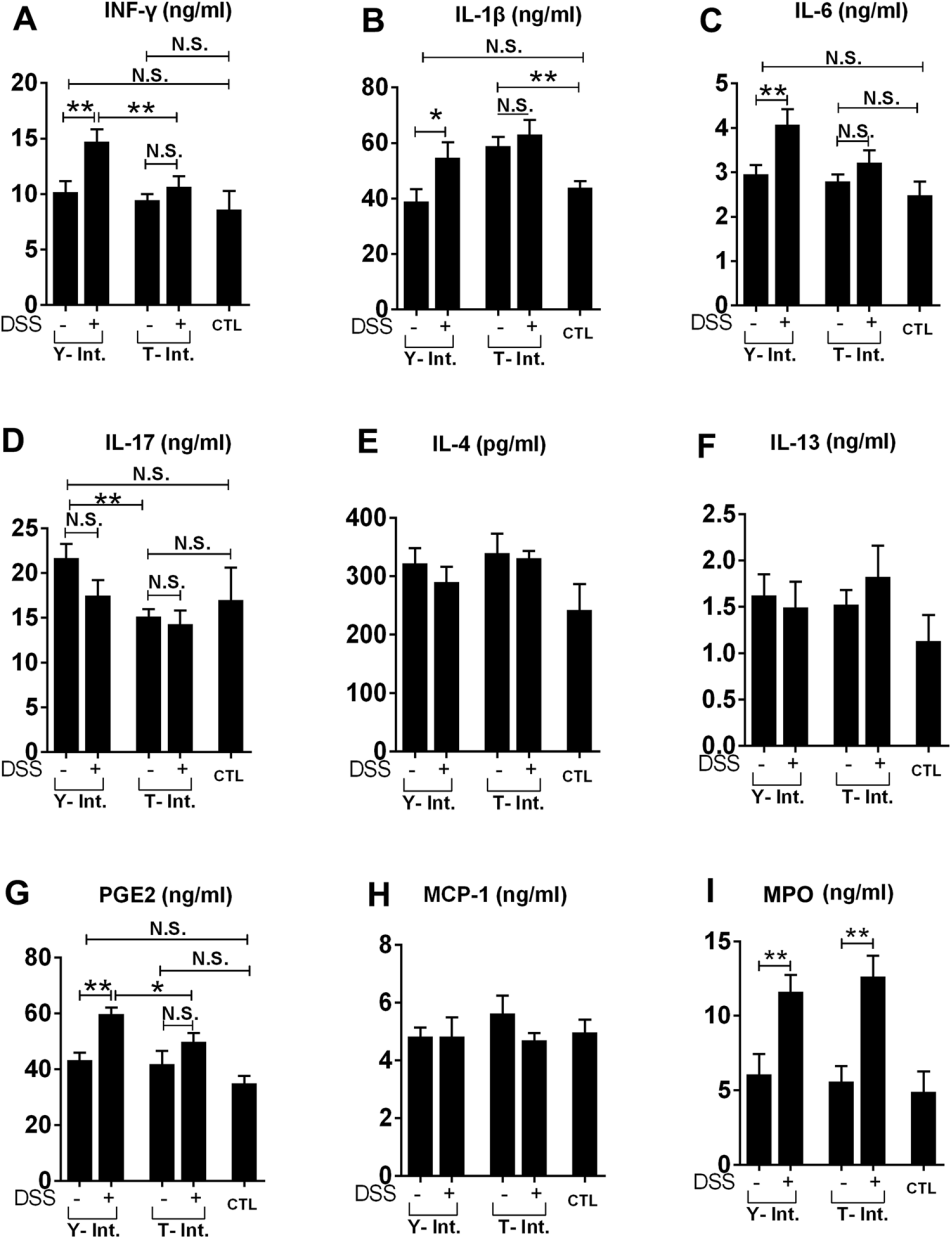
Quantitation of cytokines in colonic tissues from the piglets by ELISA. The faecal microbiota of the Yorkshire and Tibetan pigs was transplanted in healthy commercial hybrid newborn piglets to establish the “Yorkshire-intervened” (Y-int.) and “Tibetan-intervened” (T-int.) porcine models. CTL, control group (without DSS and FMT). The results are presented as the mean ± the SEM. Statistical significance was estimated using two-way ANOVA with DSS treatment and faecal microbiota transplantation as the independent variables. *P < 0.05; **P < 0.01. N.S., no significant difference. Each n = 5.

The quantitation of the colonic INF-γ, IL-1β, IL-4, IL-6, IL-13, IL-17, PGE2, MCP-1 and MPO levels is shown in Fig. 6. The inflammatory markers INF-γ, IL-1β, IL-6 and PGE2 were promoted by DSS administration only in pigs that received the “Yorkshire microbiota”. Conversely, these markers were not affected by DSS in the Tibetan microbiota-intervened group. Furthermore, the colonic INF-γ and PGE2 levels in the Tibetan group were maintained at the same levels as the control group even after DSS administration. However, the microbiota from the Tibetan but not Yorkshire pigs elevated IL-1β. The two-way ANOVA showed that microbiota intervention alone significantly enhanced the amount of IL-10 but decreased the IL-17 level. The IL-4, IL-13 and MCP-1 levels were not affected by DSS or the microbiota, and MPO was promoted only by DSS. No significant interaction effects between DSS and the microbiota were found for any of the analysed cytokines or chemokines.

#### mRNA expression levels of TLRs, NLRs and associated molecules

**Figure 7:**
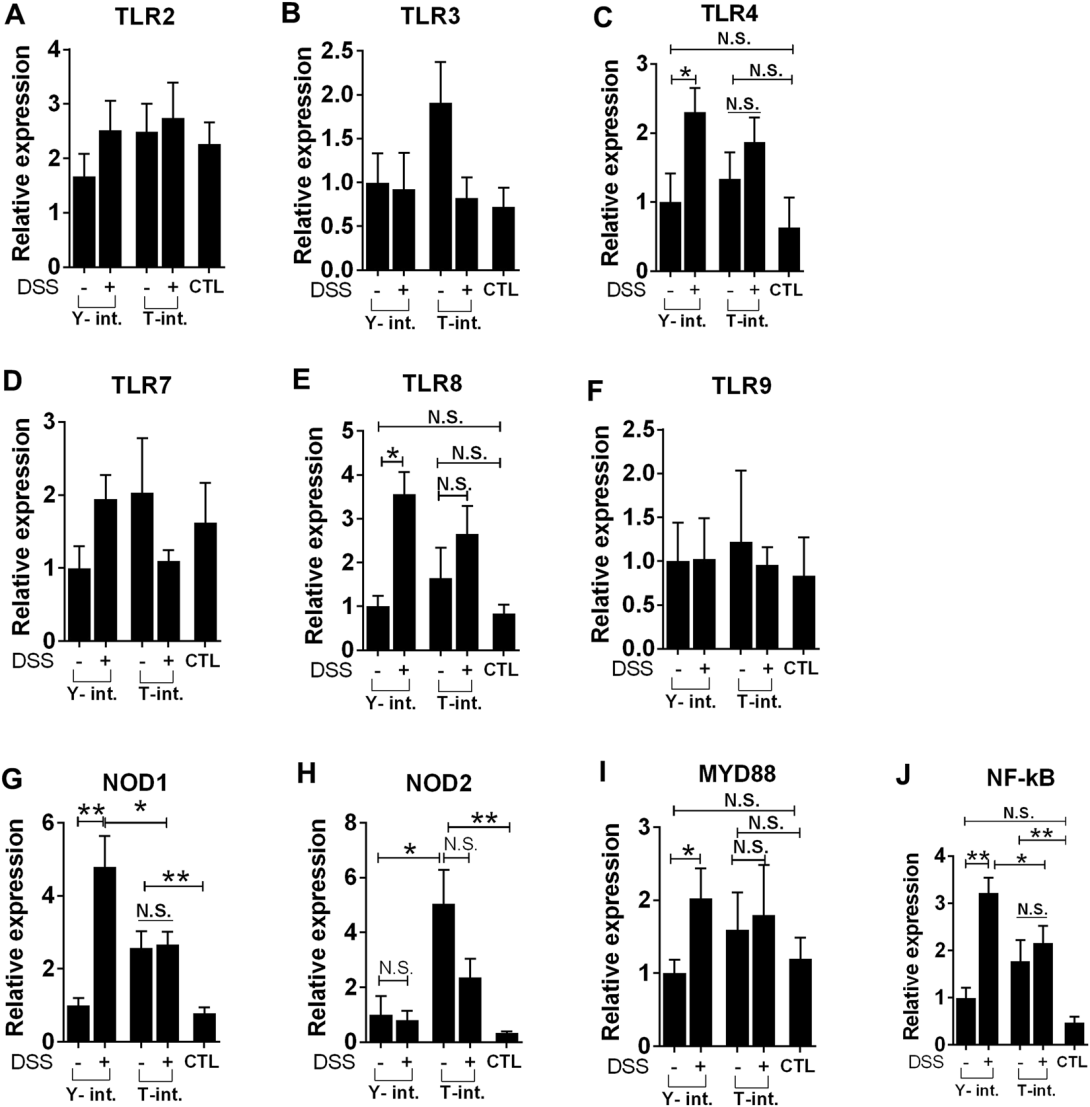
Relative quantities of TLR, NLR and associated molecule mRNAs in intestinal tissues from microbiota-transplanted piglets (each n = 6). The faecal microbiota of the Yorkshire and Tibetan pigs was transplanted in healthy commercial hybrid newborn piglets to establish the “Yorkshire-intervened” (Y-int.) and “Tibetan-intervened” (T-int.) porcine models. CTL, control group (without DSS and FMT). Total RNA was prepared from the colons as described in the “Materials and Methods”. Relative mRNA expression was quantified by qRT-PCR. The results are presented as the mean ± the SEM. *P < 0.05; **P < 0.01. N.S., no significant difference.

The mRNA expression measurements in the colonic tissues of the recipients are summarized in Fig. 7. The administration of DSS significantly elevated TLR4, TLR8, NOD1, MYD88 and NF-κB expression only in the Yorkshire group. The Tibetan pig faecal transplantation alone elevated NOD1 gene expression compared with that in the control (3.3-fold) and the Yorkshire group (2.6-fold). However, DSS administration significantly lowered the NOD1 expression level in the Tibetan group compared to that in the Yorkshire group, indicating that although the “Tibetan microbiota” was likely to activate some of the PRRs under normal physiological conditions, once under the colitis condition, this microbiota suppressed the activation of PRRs and their downstream molecules, including MYD88 and NF-κB. The “Tibetan microbiota” also promoted NOD2 expression, which was not affected by DSS. Furthermore, the other PRRs measured in this study (TLR2, TLR3, TLR7 and TLR9) were not affected by DSS or FMT. The two-way ANOVA showed significant interactions between the DSS infusion and FMT with TLR8 (*P* = 0.04), NOD1 (*P* = 0.007) and MYD88 expression (*P* = 0.04).

## Discussion

Our results show the impact of genetic factors on the porcine gut microbiota composition. Recent metatranscriptomic studies suggest that many species in the gut microbiota may be capable of performing autologous functions for the host^2^. This possibility has given rise to the hypothesis that the strong disease-resistance characteristic of Tibetan pigs is somehow associated with their unique microbial composition. The microbiome analysis in the present study revealed a significant difference in the abundance of Bacteroidetes in the faeces of these two pig strains. The proportion of Bacteroidetes in the faeces of the Yorkshire pigs was almost 50 percent higher than the proportion in the Tibetan pig faeces. At the genus level, the discrepancies are illustrated by the abundance of *Prevotella*, which has been reported to be associated with ulcerative colitis^21^. Meanwhile, the proportion of *Fibrobacter* in the faeces of the Tibetan pigs was more than 5 times higher than the proportion in the Yorkshire pig faeces. *Fibrobacter* spp. have been identified as an important cellulose-degrading bacteria provides health benefits to the host^22, 23^. Similarly, *Lactobacillus* strains are known as probiotics for their anti-inflammatory capacity,^24^ and we found the relative abundance of the *Lactobacillus* was much higher in the FT than in the FY. Taken together, the Tibetan pigs are more likely to have “healthier” microbiota communities compared to the Yorkshire pigs.

However, surprisingly, the proportion of spirochaetes in the faeces of the Tibetan pigs was more than triple the proportion in the Yorkshire pig faeces. Some groups of spirochaetes, such as *Serpulina hyodysenteriae* and *Brachyspira pilosicoli*, are aetiological agents of porcine intestinal spirochaetosis, which causes swine dysentery (a type of severe colitis)^25, 26^. At the genus level, the discrepancies are illustrated by the abundance of *Treponema*, which has been reported to cause chronic inflammatory disorders in mice^27^. The microbiota has the potential to exert both pro- and anti-inflammatory actions. Although opportunistic pathogens elicit immune responses that result in tissue damage during infection, some symbiotic bacterial species have been shown to prevent inflammatory disease during colonization^26, 27^. Interestingly, the “normal” microbiota also contains microorganisms that have been shown to induce inflammation under particular conditions^2^, suggesting that a certain bacterium (*Treponema* in this study) may play different roles to the host immune system depending on the composition of the bacterial community in the gut.

Some phenotypes are transmissible through microbiota transplants from one individual to another^28^. We demonstrated in the present study that the expression levels of NF-κB and some of the NLRs and TLRs as well as the levels of several downstream pro-inflammatory cytokines were lower in the guts of the Tibetan pigs than in the Yorkshire pigs. However, these immunological characteristics of the Tibetan pigs were not reflected in the piglets that received their faecal microbiota; instead, the NOD1/2 signalling of the recipients was activated by the “Tibetan microbiota”. The culprit might be the distinct “pathogens” in the faeces of the Tibetan pigs because infection with spirochaetes has been reported to activate the PRR signalling pathways^29, 30^. Nevertheless, the responses to foreign organisms were not sufficiently strong to cause clinical or histological activity in this study. Moreover, we showed that the commercially bred piglets accepted the “Yorkshire microbiota” much more “easily” than the “Tibetan microbiota” without triggering an observable immune response, perhaps because the cross-bred piglets shared close genetic backgrounds with the Yorkshire pigs.

However, the situation was reversed when experimental colitis was induced. To evaluate the potential benefits of supplementation with the bacteria in an inflammatory model, DSS was orally administered to two groups of pigs that received the faecal matter of Yorkshire and Tibetan pigs. Compared with the Yorkshire group, the pigs inoculated with the Tibetan microbiota showed strong resistance to the inflammation induced by DSS, with less severe colonic haemorrhaging and milder histological disease features. A notable difference between these two groups was observed in TLR and NLR signalling, which was significantly activated against DSS in the pigs that received the “Yorkshire microbiota”; these piglets produced higher levels of cytokines such as IFN-γ, IL-6, IL-1β and PGE2, resulting in increased IgA^+^ plasma cell and macrophage numbers and the CD4/CD8 ratio in the gut. These changes were not observed in the Tibetan group because the immune system retained self-tolerance (or “ignorance”) to the pro-inflammatory factors.

A growing number of studies have shed light on the dual character of TLR signalling regarding its anti- and pro-inflammatory functions. Commensal bacteria are recognized by TLRs under normal steady-state conditions, and this interaction plays a crucial role in the maintenance of intestinal epithelial homeostasis. Meanwhile, activation of TLRs by commensal microbiota is critical for protection against gut injury and associated mortality^7^. However, autoimmune inflammation is closely associated with enhanced TLR/NLR expression in inflamed tissues, indicating that this inflammation is mediated by innate immune responses through PRRs, such as TLRs and NLRs^22^. The findings in the present study may provide an explanation for the different outcomes (normal or inflammatory) following activation of NLR/TLR signalling by FMT/DSS, respectively.

Experimental colitis is a well-recognized autoimmune disorder^23^. Eliminating invading pathogens is important for the maintenance of the delicate balance in the immune system and self-tolerance to avoid autoimmunity, with the commensal bacteria in the gastrointestinal tract playing a critical role by regulating immune homeostasis^22^. Growing evidence suggests that changes in the gut microbiota composition are instrumental for the development of autoimmune diseases^24, 30, 31^, thus, a strong connection most likely exists between the faecal microbiota used in this study and the maintenance of self-tolerance to avoid autoimmunity. However, how these “immune ignorance” effects are achieved in the Yorkshire piglets by the microbiota from the Tibetan pigs is unclear. Further research is needed to unravel the microbiome/immune system/autoimmune disease axis and to provide insights into the causes and potential cures of autoimmune diseases by elucidating the molecular mechanisms whereby symbiotic microbes affect immune reactions to self-antigens.

In conclusion, this study demonstrates a large distinction in the faecal microbiota composition of two distinct pig lines representing poor (Yorkshire) and strong (Tibetan) disease resistance capabilities and reveals the contribution of the microbiota to the regulation of innate immune responses using faecal microbe transplantation technology. Taken together, these data demonstrate that the Tibetan pigs may harbour a specific gut microbiome that activates PRRs, such as NOD1/2, and results in immune activation of the recipient via the recruitment of immune cells kept at a non-inflammatory level, which seemed to be important for experimental colitis resistance. These findings fill an important gap in the domain of cross-host immunophenotype transmission and provide new approaches to improve anti-inflammatory functions through microbiota transplantation.

## Materials and Methods

### Animals, experimental design and induction of intestinal inflammation

All methods in this study were performed in accordance with the Guide for the Care and Use of Laboratory Animals prepared by the Institutional Animal Care and Use Committee of Sichuan Agricultural University (SICAU), and all the animal protocols were approved by the Animal Care and Use Committee of SICAU under permit number DKY-B20131705. Five female Yorkshire and five female Tibetan pigs from the Sichuan Reservation Farm were used in the current research as faecal donors and research models for the experiment in Part I. The donors were similar in age, were raised from birth in the same environment and were fed the same diet without antibiotics. Routine diagnoses for specific pathogens, including viruses and parasites, were performed before the experiment to ensure that the donors met the SICAU specific pathogen-free standard for pigs. 5 groups of commercial hybrid newborn piglets, which had a genetic background much closer to the Yorkshire pig than to the Tibetan pig, were used for the experiment in Part II. The pigs were segregated from their mothers immediately after birth, surface-sterilized and randomly allotted to 4 groups matched for body weight and gender as follows: Groups (G) 1 and G2 received a faecal suspension from the Yorkshire pigs and G3 and G4 received a faecal suspension from the Tibetan pigs. G5 (control group) received only sterile PBS without faeces. There were six piglets in each group. The piglets were fed artificially with milk substitutes for the first 15 days of the experiment and then switched to solid commercial foodstuffs until the end of the study. The same diet was used for all groups throughout the process. The pigs in each group were housed in separate rooms with ventilation and disinfectant installations at the entrances to avoid microbial cross-contamination. On day 50, acute colitis was induced in the pigs in G2 and G4 using dextran sulphate sodium (DSS, MW: 36,000–50,000, MP Biomedicals, USA) administration. The dose was set according to the study by Bassaganya *et al*.^32^. The control (CTL) group of piglets received no DSS neither.

### DNA extraction and microbiota analysis

Total DNA was isolated and purified using the QIAamp DNA Stool Mini Kit (Qiagen, GmbH Hilden, Germany) modified to contain a bead-beating step. The concentration and purity of the extracted genomic DNA were measured using the NanoDrop ND-1000 Spectrophotometer (NanoDrop, Germany). The integrity of the extracted genomic DNA was determined by electrophoresis on a 1% (w/v) agarose gel. Sequencing and bioinformatics analyses were performed by BGI (Shenzhen, China). Prior to high-throughput sequencing, a DNA library was prepared as previously described^33^. Briefly, the DNA extracted from the faecal samples was used as a template to amplify the hypervariable V3 region of the 16S ribosomal RNA gene. Libraries were normalized and pooled, and sequenced on the MiSeq system using v3 reagents. The primers contained a base pair sequence complementary to the V3 and V4 regions as well as Illumina adaptors and molecular barcodes as previously described^21^. The resulting amplicons were gel purified, quantified, pooled and sequenced using the 250-bp paired-end read strategy on the Illumina HiSeq 2000 platform.

The raw sequence reads were filtered and assembled. Sequences with contaminated adapters, an undetermined nucleotide, or low complexity were removed. Additional read preprocessing included 1) filtering of low-quality reads and 2) removal of primer regions. The resulting sequences were clustered into operational taxonomic units (OTUs) using USEARCH drive5 at 97% sequence similarity. Chimeric OTUs were removed using UCHIME v4.2. Representative sequences for each OTU were picked and aligned using QIIME 1.8. The Ribosomal Database Project classifier v2.2 was used to assign a taxonomic rank to each sequence in the representative set. The relative abundance of each OTU was examined at different taxonomic levels. For β-diversity analyses, the number of reads per sample was randomly subsampled to 30,000 to minimize biases caused by sequencing depth. Genetic diversity calculations and taxonomic community assessments were performed using QIIME 1.8 scripts.

### Faecal microbiota transplantation

To acquire representative faecal material for each genotype, fresh faeces were collected from 5 pigs of each breed after 12 hours of fasting. The fresh faecal samples derived from 5 pigs within the same breed were mixed and used as the faecal inoculum. The stool suspension was prepared as previously described by Zeng *et al*.^34^. Piglets in each group were colonized with 10 ml of the faecal suspension by intragastric administration daily for the first 3 days of the experiment. Then, the piglets received 10 ml of the faecal suspension every 2 days from days 4–15 and 20 ml every 5 days from days 16–48.

### Clinical evaluation and necropsy procedures

For the donors, spontaneously excreted faecal samples were collected from 5 pigs of each breed and immediately stored at −80 °C prior to DNA extraction for the microbial genome analysis. Then, all the pigs were sacrificed under anaesthesia. Ileum and colon tissue samples were obtained and immediately stored at −80 °C for the gene expression and cytokine analyses. For the piglets, clinical signs of disease were monitored on a daily basis following DSS challenge. The disease activity index was calculated using a modification of a previously published compounded clinical score^35^. The body weight and stool consistency were determined daily during the induction. The faecal occult blood (FOB) index was based on the results obtained using FOB test paper strips (colloidal gold method, JHK Biotech). The clinical score was assessed by trained individuals blinded to the treatment groups according to the standards described by Siegmund *et al*.^36^ The pigs were sacrificed under anaesthesia on day 56 of the experiment. Spiral colon sections were obtained, fixed in 10% buffered neutral formalin, embedded in paraffin for immunohistochemical analysis, sectioned (6 mm) and stained with haematoxylin and eosin (H&E) for histological examination. Ileum and colon tissue samples were immediately stored at −80 °C for the gene expression and cytokine analyses.

### Cytokine and chemokine production assay

The colons were excised, flushed with PBS, and opened along the longitudinal axis. Thereafter, 5-mm^2^ punch biopsies were excised and incubated for 24 h in a 24-well plate with RPMI 1640 medium (Life Technologies, Inc.) supplemented with 10% foetal bovine serum (FBS). The supernatants were collected and kept at −80 °C prior to assessment of the cytokine/chemokine levels. The TNF-α, IFN-γ, IL-1β, IL-4, IL-6, IL-10, IL-12, IL-17, PGE2, and MCP-1 levels were measured in the supernatants using the ELISA Duo-Set kit according to the manufacturer’s instructions (R&D Systems, Minneapolis, MN, USA). MPO was analysed from tissue homogenates using a Myeloperoxidase Assay Kit (Jiancheng, NJ, China).

### Measuring the regulation of gene expression by real-time quantitative reverse transcriptase polymerase chain reaction (qRT-PCR)

Colonic samples (0.1 g) were homogenized in 1 ml of the TRIzol reagent (Invitrogen), and total RNA was extracted following the manufacturer’s instructions. The concentration and purity of the RNA were analysed spectrophotometrically (Beckman Coulter DU800; Beckman Coulter Inc.); the OD260:OD280 ratio (where OD is the optical density) ranged from 1.8 to 2.0 for all samples. The RNA integrity was measured by formaldehyde gel electrophoresis, with the 28S:18S ribosomal RNA band ratio ≥1.8. The RNA samples were reverse transcribed into complementary DNA using the PrimeScripte^TM^ RT reagent kit (Takara) according to the manufacturer’s instructions. The primers commercially synthesized by Life Technologies Limited are listed in Table S2. Real-time PCR for the quantification was performed on the Opticon DNA Engine (Bio-Rad) using SYBR Green PCR reagents (Takara). β-Actin was chosen as the reference gene transcript, and the relative expression ratio of the target gene compared with the reference gene was calculated as described previously^37^. Each standard and sample was run simultaneously in duplicate on the same PCR plate, and the average of each duplicate value expressed as the number of copies was used for the statistical analysis.

### Immunohistochemistry

The avidin–biotin–peroxidase complex (ABC) method^38^ was used on paraffin-embedded colon sections in this study. The tissues were dewaxed and rehydrated, and endogenous peroxidase activity was blocked by incubation of the sections with 0.3% hydrogen peroxide in methanol for 30 min at room temperature. The tissue sections were digested with microwave pretreatment in 10 mM Tris–EDTA (pH 9.0) (MWE) for 5–15 min in a domestic HF 2640 (Siemens, Germany) at maximum power (750 W). Then, all tissue sections were incubated with 10% normal goat serum (Vector Laboratories, Burlingame, CA, USA) for 30 min at room temperature. The primary antibodies (Table S3) were applied overnight at 4 °C. Preliminary experiments were performed to determine the optimal dilutions of the different antibodies. A biotinylated goat or rabbit anti-mouse IgG (Vector) for the monoclonal antibodies or anti-rabbit IgG (Vector) for the polyclonal antibodies (both diluted 1:200) was applied as the secondary reagent. An ABC complex (Vector) diluted 1:50 was applied as the third reagent. The sections were incubated for 1 min with 0.035% 3,3’-diaminobenzidine tetrahydrochloride (Sigma) in TBS containing 0.1% hydrogen peroxide. Then, the sections were rinsed in running tap water for 10 min, dehydrated in a graded ethanol series, cleared in xylene, mounted in Eukitt (Merck Eurolab, Albertslund, Denmark), and examined under an Imager A1 microscope (Zeiss) equipped with a digital camera (AxioCam MRc 5, Zeiss).

### Quantitative and statistical analyses

The results are shown as the means ± the SEM. The statistical analysis was performed with Student’s t-test or two-way ANOVA unless otherwise indicated. Significance was accepted at *P* < 0.05. For the quantitative analysis, all samples were evaluated at 200x magnification using an Imager A1 microscope (Zeiss) equipped with a digital camera (AxioCam MRc 5, Zeiss) and an image analyser (Image-Pro Plus^TM^, Media Cybernetics, Silver Spring, MD, USA). The integrated optical density (IOD) was calculated for arbitrary areas in 10 fields with a standard area (0.04 mm^2^/field) per slide. Five sections were analysed per animal. The data obtained from the different experimental groups were analysed and scored blindly.

## Electronic supplementary material

Supplementary Information
