# Supplementary material for: Early Gut Microbiota Intervention Suppresses DSS-Induced Inflammatory Responses by Deactivating TLR/NLR Signalling in Pigs
Source: Sci Rep. 2017 Jun 12;7:3224. doi: 10.1038/s41598-017-03161-6 (PMC5468271; doi:10.1038/s41598-017-03161-6)
Supplement: Supplementary file 1 — Supplementary Information [file 41598_2017_3161_MOESM1_ESM.pdf]

Early Gut Microbiota Intervention Suppresses DSS-Induced Inflammatory Responses by Deactivating TLR/NLR Signalling in Pigs

Yi Xiao[a], Honglin Yan[a], Hui Diao[a], Bing Yu, Jun He, Jie Yu, Ping Zheng, Xiangbing Mao, Yuheng Luo and Daiwen Chen*

Key Laboratory of Animal Disease-Resistance Nutrition, Animal Nutrition Institute, Sichuan Agricultural University, Yaan, Sichuan, China, 625014.

Short Title: Microbiota Suppresses Inflammatory Responses

*Address correspondence to Daiwen Chen, dwchen@sicau.edu.cn

[a]Y.X., H.Y. and H.D. contributed equally to this work.

Supplementary information

Table S1 Raw reads, sequences and OTUs among four groups

Group	Raw reads	Clean Reads	OTUs
Y1	62267	60492	839
Y2	56742	54416	703
Y3	61579	59733	779
Y4	52202	51241	624
Y5	56946	55436	775
T1	59839	57261	869
T2	57601	50576	837
T3	42732	40134	639
T4	61093	59390	730
T5	66076	58597	818

Y, Yorkshire pig; T, Tibetan pig; OTUs, operational taxonomic units.

Table S2 PCR primer pairs for the amplification of porcine mRNA

Primer	Nucleotide sequence (5'-3')	Product size (bp)	GenBank accession number	Annealing temperature (℃)
β-Actin				
Forward	TCTGGCACCACACCTTCT	114	NM_001101	57
Reverse	TGATCTGGGTCATCTTCTCAC			
TLR2				
Forward	CGGCTTCCAAGGATGGAGAAA	114	AY392087.1	60
Reverse	CAATCCCCAAGACCCATGCT			
TLR4				
Forward	TTACAGAAGCTGGTTGCCGT	152	GQ304754	60
Reverse	TCCAGGTTGGGCAGGTTAGA			
TLR9				
Forward	CCAGCCAGACCCTTTGGAGA	174	NM_213958.1	60
Reverse	GGAGAGTAAGGAGAGGCTGGT			
TLR3				
Forward	TGGAAAAAGGAATGGCCAGC	218	NM_001097444.1	60
Reverse	ACAAGGCAAACTCCTGCTCA			
TLR7				
Forward	CAATGGTCCCTGAGCGTTTG	152	EF583901.1	60
Reverse	AGCCTGGTTGAAGACAGCAG			
TLR8				
Forward	AGAGCTGCTAATTGGTGCCTT	214	NM_214187.1	60
Reverse	AGGCAGGTCAGGAGCAAAAA			
NF-κB				
Forward	TTCTGGACCGCTTGGGTAAC	120	DQ834921.1	60
Reverse	CACCGTTGGGGTGGTTGATA			
MYD88				

Forward	GCTCTTCCTAAACGTGCGGA	241	AB292176.1	60
Reverse	TCGGCAGTCCTCTTCAATGC			
NOD1				
Forward	TCAACACCGATCCAGTGAGC	237	NM_001114277.1	60
Reverse	TGAAAATGGTCTCGCCCTCC			
NOD2				
Forward	GTGCCTCCCCTCTAGACTCA	191	NM_001105295.1	60
Reverse	ACGAACCAGGAAGCCAAGAG			

Table S3 Primary antibodies used in the immunohistochemical studies

Antibody	Clonality	Dilution	Source*
CD4	Monoclonal	1:200	BBEL
CD8	Monoclonal	1:100	BBEL
IgA	Polyclonal	1:200	BBEL
MAC387	Polyclonal	1:200	BBEL

* BBEL, Boster Biological Engineering Lab, Wuhan, China.

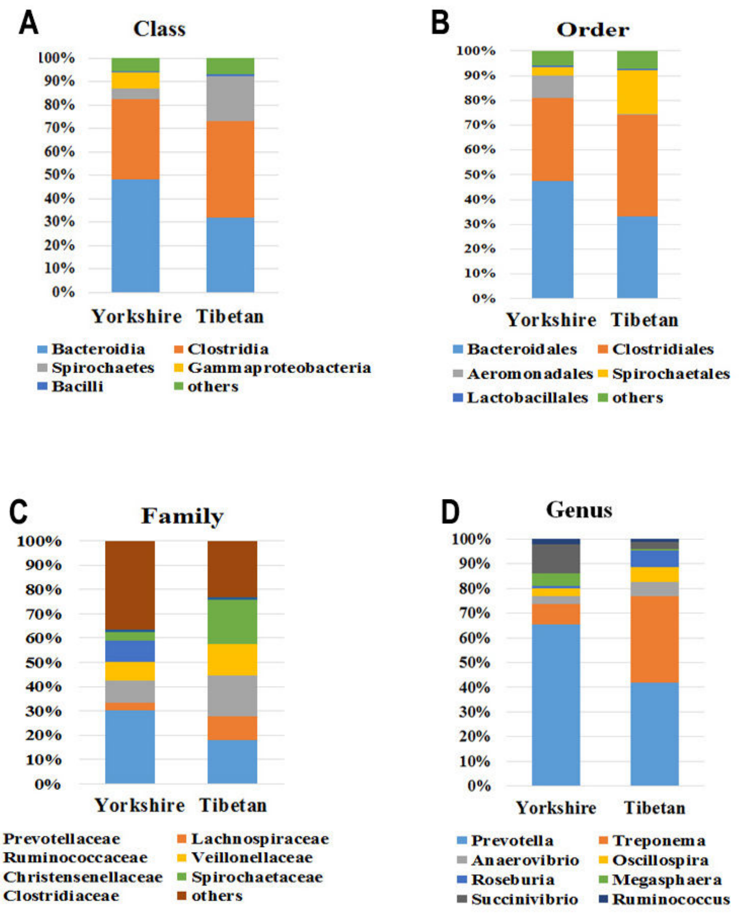

Figure S1 Relative abundance of major taxonomic (A) classes, (B) orders, (C) families and (D) genera in two strains from Yorkshire pigs and Tibetan pigs. Groups within the same bacterial phylum are indicated by different shades of the same color. Taxa with a mean relative abundance > 1% are shown (n = 5 for each strain).
